# Dorsal Horn of Mouse Lumbar Spinal Cord Imaged with CLARITY

**DOI:** 10.1155/2020/3689380

**Published:** 2020-08-14

**Authors:** Gulgun Sengul, Huazheng Liang, Teri M. Furlong, George Paxinos

**Affiliations:** ^1^Department of Anatomy, School of Medicine, Ege University, Bornova, Izmir 35100, Turkey; ^2^Neuroscience Research Australia, 139 Barker St, Randwick, Sydney NSW 2031, Australia; ^3^Department of Neurology, Translational Research Institute of Brain and Brain-Like Intelligence, Shanghai Fourth People's Hospital Affiliated to Tongji University School of Medicine, Shanghai 200081, China; ^4^School of Medical Sciences, The University of New South Wales, Kensington, NSW 2052, Australia

## Abstract

The organization of the mouse spinal dorsal horn has been delineated in 2D for the six Rexed laminae in our publication *Atlas of the Spinal Cord: Mouse, Rat, Rhesus, Marmoset, and Human*. In the present study, the tissue clearing technique CLARITY was used to observe the cyto- and chemoarchitecture of the mouse spinal cord in 3D, using a variety of immunohistochemical markers. We confirm prior observations regarding the location of glycine and serotonin immunoreactivities. Novel observations include the demonstration of numerous calcitonin gene-related peptide (CGRP) perikarya, as well as CGRP fibers and terminals in all laminae of the dorsal horn. We also observed sparse choline acetyltransferase (ChAT) immunoreactivity in small perikarya and fibers and terminals in all dorsal horn laminae, while gamma aminobutyric acid (GABA) and glutamate decarboxylase-67 (GAD67) immunoreactivities were found only in small perikarya and fibers. Finally, numerous serotonergic fibers were observed in all laminae of the dorsal horn. In conclusion, CLARITY confirmed the 2D immunohistochemical properties of the spinal cord. Furthermore, we observed novel anatomical characteristics of the spinal cord and demonstrated that CLARITY can be used on spinal cord tissue to examine many proteins of interest.

## 1. Introduction

The major characteristics of the neurons of each of the spinal dorsal horn laminae have been studied using standard histological, histochemical, and immunohistochemical staining methods. Our mouse spinal cord atlases published in 2009 and 2013 have provided a detailed map of the spinal cord laminae and their neurochemical content using the marker acetylcholinesterase and immunohistochemistry for substances such as calbindin (Cb), calcitonin gene-related peptide (CGRP), calretinin (Cr), choline acetyltransferase (ChAT), enkephalin, neurofilament protein (SMI-32), and neuronal nuclear protein (NeuN) [1, 2].

The spinal dorsal horn is morphologically heterogeneous, but there is an intrinsic organization, so that zones of regularity or laminae can be identified. The ten laminae in the spinal cord gray matter based on cellular shape, size, and density were first described by Rexed in the cat [3, 4], followed by studies in the mouse [2, 5, 6], rat [2, 7, 8], marmoset, rhesus monkey, and human [2]. Six of these laminae (laminae 1-6) are in the dorsal horn. Neurons within a lamina often show distinct dendritic architecture, chemoarchitecture, and patterns of connections, as well as different functions. These neurons receive a variety of neurotransmitter inputs and release a variety of neurotransmitters that show different patterns of distribution that is often similar across different species.

Transverse sections of the spinal cord show the laminar distribution of the neurons, while sagittal sections are useful for the determination of the morphology of neurons and the distribution of their dendritic and axonal processes. Until recently, the only way to visualize the spinal cord in 3D was to obtain serial sections and make a reconstruction of them. However, tissue sectioning results in the breaking of axons and dendrites. Consequently, the broken axons and dendrites are difficult to be accurately realigned in 3D reconstruction.

CLARITY is a relatively novel technique developed by Karl Deisseroth and his colleagues that turns the brain transparent using the detergent sodium dodecyl sulfate by removing lipids that normally block the passage of light [9]. Unlike traditional histological methods where the tissue is sectioned for imaging, 3D imaging of the entire cleared tissue allows examination of labeled cells and molecules in the entire specimen, allowing marking and visualization of long-range projections and subcellular structures. The other advantage of CLARITY is the application of lipophilic dyes to trace neurons and their projections in the cleared tissue [1].

Thus far, the only studies using CLARITY in the spinal cord have examined the distribution of neuronal calcium-binding proteins 1 and 2 (NECABB1/2) [10] and serotonergic fibers [11]. In this study, we aimed to show the cyto- and chemoarchitecture of the mouse spinal cord dorsal horn with many protein markers, i.e., calretinin, glycine, nitric oxide, CGRP, ChAT, GAD67, and serotonin 3D using CLARITY for the first time.

## 2. Materials and Methods

### 2.1. Animals

The animal work was approved by the Animal Care and Ethics Committee (ACEC) at The University of New South Wales (approval number 14/94A). Experiments were conducted on C57BL/6J (*n* = 12) male mice (http://jaxmice.jax.org/strain/013636.html) and transgenic (ChAT:eGFP, *n* = 3) male mice (https://www.jax.org/strain/007902) expressing enhanced green fluorescent protein in cholinergic neurons.

### 2.2. Tissue Clearing

Mice were anaesthetized with a lethal dose of pentobarbital solution (0.1 ml, 200 mg/ml) and perfused with ice-cold hydrogel solution containing 4% acrylamide, 0.05% Bis, 0.025% VA-044 initiator, and 4% PFA in PBS, following the protocol of Chung et al. [9]. After removal, the spinal cord was cut into 2-3 mm segments and kept in 5 ml plastic tubes at 4°C overnight on a rotator. Subsequently, the tissue was degassed by replacing the air with nitrogen in a fume hood for 10-15 min. The tissue was then transferred to a 37°C oven for incubation on a rotator. After the solution became a hydrogel, the tissue was removed from the gel and washed with the clearing solution (0.2 M boric acid, 4% sodium dodecyl sulfate, pH 8.5) for 7-10 d until the tissue became transparent.

### 2.3. Immunofluorescence Staining

The following procedure was modified from a protocol by Tomer et al. [12]. After washing the lumbar cord tissue with PBST (0.1% Triton X-100) every 6 h for 24 h, the tissue was incubated in primary antibody solutions (1 : 100) for 3 d on a rotator at 37°C. For primary antibodies, we used rabbit anti-calretinin (Merck Millipore, AB5054), rabbit anti-CGRP (Merck Millipore, Cat# AB15360), goat anti-ChAT (Merck Millipore, Cat# AB144P), rabbit anti-serotonin (Sigma, Cat# S5545), sheep anti-glycine transporter 2 neuronal (Merck Millipore, Cat# AB1771), mouse anti-GAD67 (clone 1G10.2; Merck Millipore, Cat# MAB5406), rabbit anti-nitric oxide synthase (anti-NOS, Merck Millipore, Cat# AB5380), and rabbit anti-GABA (Sigma, Cat# A2052). The tissue was subsequently washed with PBST every 6 h for 24 h and incubated in secondary antibodies, Alexa Fluor 594 conjugated goat anti-rabbit IgG (Life Technologies, Cat# A-11012), Alexa Fluor 594 conjugated goat anti-mouse IgG (Life Technologies, Cat# A-11032), Alexa Fluor 594 conjugated donkey anti-goat IgG (Life Technologies, Cat# A-11058), or Alexa Fluor 594 conjugated donkey anti-sheep IgG (Life Technologies, Cat# A-11016) for 3 days at 37°C, at 1 : 100 dilution each, before they were washed again with PBST. After 24 h, the tissue was put into 85% glycerol for homogenizing the refractive index before imaging.

Spinal cord tissues were imaged with a Leica TCS SP5 multiphoton microscope (Leica Microsystems GmbH, Wetzlar, Germany) using 20x and 63x objectives. Leica TCS SP5 II has four lasers with the following lines for excitation: diode 50 mW with 405 nm emission line, “blue” argon multiline 65 mW with 458/476/488/514 nm emission lines, “yellow” diode 20 mW with 561 nm emission line, and “red” 10 mW with 633 nm emission line. Five channels, including a TLD brightfield detector, an internal detector channel (PMT), and three internal GaAsP detectors (HyD), were available to use. For 20x imaging, HC PL APO 20x/0.70 IMM CORR CS, H_2_O/glycerol/oil, objective numerical aperture was 0.70, resolution *XY*: 279, resolution *Z*: 1284, and free working distance: 260. For 63x imaging, HCX PL APO 63x/1.30 GLYC CORR CS 21°C, objective numerical aperture was 1.3, resolution *XY*: 160, resolution *Z*: 335, and free working distance: 280. IC Prisms: C; C1-P; C1; Cond. Prism DIC: K3, K6, 405; correction optic: L1, and UV correction optic: L1. IC Prisms: C; C1-P; C1, Cond. Prism DIC: K3, K6, 405 correction optic: L1, and UV correction optic: L1. The image was scanned at 400 Hz speed at zoom 1 and resolution of 1024 × 1024 pixels (760 nm × 760 nm) with a step size of 3 *μ*m for the 20x objective and 1 *μ*m for the 63x objective. Emitted signals were collected into hybrid detectors (HD) with line accumulation of 4-8x. In the present study, the mouse lumbar cord was cut into small segments, but the imaging depth was limited by the working distance of the microscope, which is less than 300 *μ*m. 3D reconstructions were made using OsiriX Imaging Software (Pixmeo Labs., Geneva, Switzerland) as described by Rosset et al. [13].

## 3. Results

### 3.1. General Findings

**Figure 1:**
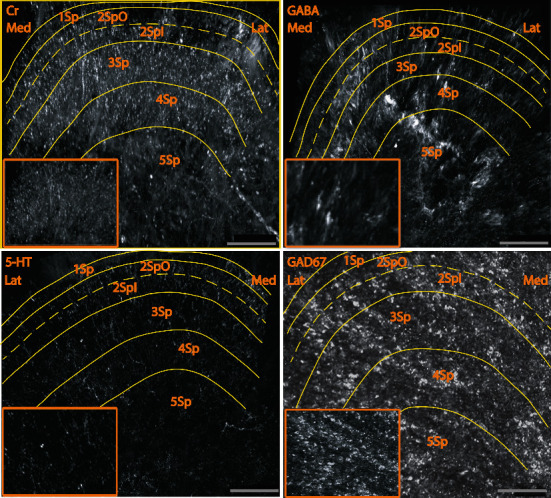
Cr (segment L1), GABA (segment L2), serotonin (segment L2), and GAD67 (segment L3) immunoreactive neurons and fibers in the mouse spinal dorsal horn. Scale bar is 100 *μ*m. Med: medial; Lat: lateral.

**Figure 2:**
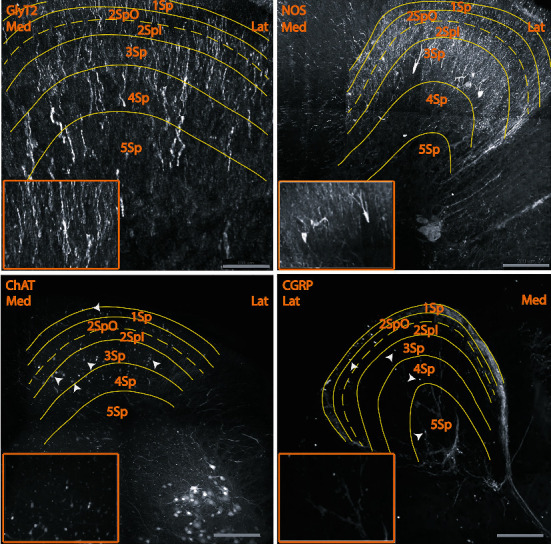
GlyT2 (segment L2), NOS (segment L2), ChAT (segment L5), and CGRP (segment L2) immunoreactive perikarya (arrowheads) and fibers in the superficial laminae of the mouse spinal dorsal horn. Scale bar: 100 *μ*m for GlyT2 and 200 *μ*m for NOS, CHAT, and CGRP. Med: medial; Lat: lateral.

This distribution of multiple proteins, including calretinin, NOS, CGRP, ChAT, GAD67, GABA, serotonin, and glycine using CLARITY. Our observations of the cyto- and chemoarchitecture of the dorsal horn were similar to earlier reports that used conventional light and fluorescent microscopy techniques (Figures 1 and 2, Online resources 1-6), thus confirming that CLARITY can be used reliably to examine spinal cord tissue. These findings are summarized in Table 1. Briefly, ChAT, CGRP, calretinin, GABA, GAD67, GlyT2, and NOS immunoreactive neurons were observed across all laminae of the dorsal horn in the depth as long as the working distance of the microscope. Serotonergic perikarya were not observed. The borders of each lamina were defined by cell size and density, using an atlas of the mouse spinal cord [1] constructed on cyto- and chemoarchitecture of Rexed's laminae.

### 3.2. Novel Findings Different from 2D Imaging

Our findings are mostly consistent with those of prior studies (as outlined in Table 1; also see Discussion for details). However, there were some novel observations for CGRP, ChAT, GABA, GAD67, and serotonin that we have identified using CLARITY technique.

Firstly, we observed numerous CGRP immunoreactive perikarya in all laminae along with CGRP fibers and terminals, which were densest in laminae 1 and 4-6 **(**Figure 2, Online resources 1**)**. Secondly, many small ChAT perikarya were also found in all laminae, and sparse ChAT immunoreactivity was found for fibers and terminals throughout the dorsal horn after ChAT antibody labeling (Figure 2, Online resources 4). Similar findings were observed in the ChAT-GFP mouse lumbar cord. Finally, GABA and GAD67 immunoreactivities were found throughout the dorsal horn in small perikarya. GABA and GAD67 immunoreactive fibers were found more densely in laminae 1-4 and attenuated in laminae 5-6. For GABA, we also found antenna-like neurons (with dendrites extending dorsally) in lamina 3 (Figure 2, Online resources 2 and 3), an observation not previously reported. Serotonergic fibers were observed in all laminae of the dorsal horn (Figure 1, Online resources 5).

## 4. Discussion

Over the last several decades, many studies have shed light on the cyto- and chemoarchitecture of the spinal dorsal horn using a variety of techniques, especially Golgi staining, intracellular injection methods, and histochemical/immunohistochemical stains. However, these require tissue sectioning and imaging of individual sections to make reconstructions in order to see cell morphology and cytoarchitecture in detail—an approach limited by loss of tissue during sectioning, mechanical distortions, and difficulties in the alignment of the images. In the present study, the cyto- and chemoarchitecture of the mouse spinal cord dorsal horn have been revealed for the first time in 3D, using a variety of immunohistochemical protein markers with unprecedented detail. CLARITY can provide more accurate representations of subtle anatomical characteristics in the spinal cord and provide 3D videos for observation. A recent study by Ertürk et al. [14] used a tetrahydrofuran-based clearing technique to visualize microglia, astrocytes, and neurons in eGFP mice and determined the cell number and axonal regeneration following spinal cord injury. However, they did not apply any immunohistochemical markers or produce data on the cyto- and chemoarchitecture of the spinal cord.

In the present study, we observed CGRP, ChAT, GAD67, GABA, serotonin, and glycine immunoreactivities in the mouse spinal cord for the first time using the CLARITY technique. Our observations with CLARITY support earlier reports on the overall density and immunohistochemical localization of spinal cord neurons in the mouse for calretinin [15], GABA [16, 17], and NOS [18, 19] and in the rat for calretinin [20, 21], GABA [22–24], glycine [25–28], and NOS [19, 29, 30]. Replicating prior studies that used standard 2D imaging, and extending these with observations in 3D using CLARITY, is important in validating the results of this technique. This suggests that future studies can move forward with CLARITY. Finally, we observed new details for CGRP, ChAT, GABA, GAD67, and serotonin as discussed below.

### 4.1. CGRP

With CLARITY, we were able to identify numerous CGRP immunoreactive perikarya in all laminae of the mouse dorsal horn. CGRP fibers and terminals were also found in all laminae, denser in laminae 1 and 4-6 (Figure 2, Online resources 1). In studies with 2D immunohistochemical staining, CGRP fibers and terminals were shown mainly in laminae 1-2 and to a lesser extent in laminae 3-4 and 6 of the mouse spinal cord [31–33]. CGRP immunoreactive perikarya were first reported by Conrath et al. [34], encountered only rarely in lamina 1, more in lamina 2, and most frequently in lamina 3 of the rat spinal cord. Tie-Jun et al. [32] observed much fewer CGRP perikarya in the mouse, only in lamina 3. Our findings suggest that, although the major origin of CGRP in the spinal cord is from afferent fibers derived from dorsal root ganglion neurons, CGRP immunoreactive perikarya throughout the dorsal horn laminae also contribute to the sensory functions of the spinal cord including nociception. Lately, it has been suggested that CGRP facilitates nociceptive transmission and maintains central sensitization in both the primary afferent sensory neurons and the second-order pain transmission neurons within the central nervous system [35, 36]. A denser distribution of CGRP fibers in lamina 4 indicates that these laminae receive more terminals from primary sensory neurons. Given that lamina 4 is recognized to receive proprioceptive input [1], this suggests a possible role for CGRP neurons of the dorsal horn in nociceptive and nonnociceptive sensory information including proprioception. CGRP immunoreactive perikarya throughout the dorsal horn laminae presumably play a role in the sensory functions of the spinal cord including nociception.

### 4.2. ChAT

With CLARITY, we observed sparse ChAT immunoreactivity in fibers and many small perikarya in all laminae of the dorsal horn of the ChAT:eGFP transgenic mice (Figure 2, Online resources ChAT). Cholinergic neurons in the midbrain do not make direct connections with the spinal cord, and this is consistent with the absence of ChAT and VAChT immunolabeling in the spinal white matter [37]. This suggests that cholinergic fibers in the dorsal horn are either derived from intraspinal cholinergic neurons or from primary sensory neurons. Intraspinal cholinergic neurons and primary sensory neurons are the primary sources of cholinergic fibers in the spinal cord. Supporting this, cholinergic neurons in the midbrain do not have any synaptic connections with the rat spinal cord neurons [37]. A dense plexus of cholinergic fibers is found in laminae 2 and 3 of the rat spinal cord [38, 39], but no cytoplasmic immunoreactivity has been shown for ChAT or vesicular acetylcholine transporter (VAChT) in the rat, mouse, and primate spinal cords [40–42]. However, some studies reported a large number of ChAT immunoreactive neurons in laminae 3-4 of the rat [38, 43]. Recently, a study on ChAT-eGFP mice also showed a low number of perikarya (2.8 ± 0.3 perikarya per 50 *μ*m thick transverse section in laminae 3-4) [44]. As well as laminae 3-4, we have observed a dense cholinergic plexus in lamina 2i and less dense in laminae 1 and 2o. The presence of ChAT perikarya beyond laminae 3-4 indicates that ChAT has a wider range of functions in the spinal cord than previously thought. For example, ChAT is likely to modulate both nonnociceptive and nociceptive sensory information including inhibition of nociceptive transmission given that these are recognized functions of laminae 1 and 2 [45].

### 4.3. GAD67 and GABA

With CLARITY, we observed GABA and GAD67 immunoreactivities in all laminae of the dorsal horn both in fibers and small perikarya. GABA and GAD67 immunoreactive fibers were dense in laminae 1-4 and moderately dense in laminae 5-6. For GABA, we also identified antenna-like neurons that have dorsally oriented dendrites in lamina 3 (Figure 1, Online resources GAD, GABA). GABA-containing neurons within the spinal dorsal horn were initially identified in laminae 1-3 of the rat using glutamate decarboxylase (GAD) immunohistochemical staining [46–49]. The majority of these cells had ventrally oriented dendrites. Also, GABA immunoreactivity was found in small fusiform, round, or multipolar-shaped perikarya mainly in laminae 1-3 and fewer in laminae 4-6 [22, 50–52] of the rat spinal cord. The present study confirms these observations and additionally demonstrates GABA neurons in laminae 5-6 using CLARITY.

### 4.4. Serotonin

In the mouse, all serotonergic cell groups of the caudal hindbrain have projections to the spinal cord [41] and play a modulatory role in the central regulation of many autonomic functions. Previously, serotonergic fibers were observed in all laminae, being most abundant in laminae 2i and 2o [1,6,52]. With CLARITY, we observed numerous serotonergic fibers in all laminae of the dorsal horn. Similarly, in a recent study, we have observed serotonergic fibers in all laminae of the dorsal horn, excluding the lateral part of laminae 2 and 4 [11].

## 5. Conclusions

With the CLARITY clearing technique, we observed the cyto- and chemoarchitecture of the mouse spinal cord in 3D for the first time for calretinin, CGRP, ChAT, serotonin, glycine, GAD67, NOS, and GABA. We confirmed prior observations of the locations of these proteins in spinal cord laminae and reported novel observations of visualization of the proteins of interest in 3D, concluding that CLARITY is a suitable technique for visualization of the spinal cord in unprecedented detail.

## Figures and Tables

**Table 1:** Distribution of CGRP, ChAT, Cr, GABA, GAD67, GlyT2, NOS, and serotonin immunoreactive perikarya in the mouse spinal cord using CLARITY.

Lamina	1	2	3	4	5	6
CGRP	++	+	+	+	+	+
ChAT	+	++	+	+	+	+
Cr	++	++	++	+	+	+
GABA	++	++	++	++	++	++
GAD67	++	++	++	++	++	++
GlyT2	++	++	++	++	++	++
NOS	++	++	++	+	+	+
Serotonin	—	—	—	—	—	—

## Data Availability

All results produced from the present study are available upon reasonable request to the corresponding author.

## Supplementary Materials

Supplementary 1 Online resources 1: distribution of CGRP-positive fibers in the dorsal horn of the mouse L2 lumbar cord. The top is dorsal, and the right is medial.

Supplementary 2 Online resources 2: distribution of GAD67-positive fibers in the dorsal horn of the mouse L3 lumbar cord. The top is dorsal, and the right is medial.

Supplementary 3 Online resources 3: distribution of GABA-positive fibers in the dorsal horn of the mouse L2 lumbar cord. The top is dorsal, and the right is lateral.

Supplementary 4 Online resources 4: distribution of ChAT-positive fibers in the dorsal horn of the mouse L5 lumbar cord. The left is dorsal, and the top is lateral.

Supplementary 5 Online resources 5: distribution of serotonin-positive fibers in the dorsal horn of the mouse L2 lumbar cord. The left is dorsal, and the top is lateral.

Supplementary 6 Online resources 6: distribution of glycine-positive fibers in the dorsal horn of the mouse L2 lumbar cord. The top is dorsal, and the right is lateral.
